# A new disclosure index for Non-Governmental Organizations

**DOI:** 10.1371/journal.pone.0191337

**Published:** 2018-02-21

**Authors:** Ayesha Nazuk, Javid Shabbir

**Affiliations:** 1 School of Social Sciences and Humanities (S^3^H), National University of Sciences and Technology (NUST), Sector H-12, Islamabad, Pakistan; 2 Department of Statistics, Quaid-i-Azam University (QAU), Islamabad, Pakistan; University of Padua, ITALY

## Abstract

Website of Non-governmental organization (NGO) is a focal medium of sharing information in response to transparency demands and addressing trust deficits between stakeholders. Many researchers have proposed accountability approaches to measure information sharing trends through websites. This article discusses a new index to measure online disclosure trends along with the theoretical properties of the index and a practical application of data from NGOs working in Pakistan. The websites have been coded in 2016. Results show that NGOs with branch offices have better disclosure scores than single-office NGOs, and international NGOs score better than local NGOs. NGOs that are more often the subject of newspaper reports have better disclosure trends.

## 1. Introduction

Non-Governmental Organizations (NGOs) have the potential to affect public policy through advocacy. Advocacy takes many forms, and can be done using one or more of the three following non-mutually exclusive strategies: direct lobbying, indirect lobbying, and media advocacy. With the introduction of the internet and the World Wide Web (WWW), websites have become an effective medium of advocacy for NGOs. Baker [1] describes the power of the Internet as follows:

You have heard about the Information Superhighway. [It is] a transnational conglomeration of computer networks—a relentlessly expanding infrastructure that’s revolutionizing communications and methods of commerce [1].

This description is justified: In 1971, the first email was sent, although the efforts to create the Internet began in the 1960s. Since then the Internet has penetrated our world, and today our lives pivot on the axis of sharing information through digital means. The introduction of the WWW in 1993 changed the way people communicate, businesses operate, education is imparted, and services are delivered. Like any other organization, NGOs are trying to make the best use of the Internet in order to create a positive image in society.

An important challenge faced by NGOs today is the increasing trust deficit on the part of its stakeholders due to rising frequency of scandals involving NGOs [2–3]. One determinant of these scandals is the low entry barrier for new NGOs [4]. These scandals can negatively influence the public image of the NGO sector as a whole, so genuine NGOs should take proactive measures by maintaining records that can ensure their credibility. The website of any NGO serves as a publicly accessible platform to share information related to its mission, activities, and performance [5]. Ebrahim [6] defines three aspects of NGO accountability: upward accountability to its donors, downward accountability to its beneficiaries, and internal accountability to its staff. The website of any NGO offers a means of exchanging information between the organization and its stakeholders, and hence it can serve the need to provide upward, downward, and internal accountability.

Murtaza [7] pointed out that there have been increasing demands for NGO accountability, and the source of such demand is either genuine concern for improvement or a hidden motive of thwarting advocacy processes. Murtaza [7] discussed the notion that NGOs’ rhetoric empowers communities by not only helping them provide services but giving value to the feedback provided by communities on the operations of these organizations. Nevertheless, current disclosures practices are indeed tilted towards upward accountability, as these organizations are more focused on satisfying their donors and funding bodies. Thus, there is a need to suggest a mechanism for disclosures for NGOs and devise a framework that gives optimum weights to all dimensions of accountability, particularly accountability to society. Websites offer a low-cost, two-way communication process to share the information expected of these organizations, including, but not limited to, financial disclosures, tangible and intangible outputs, and governance practices [4–5, 8–9].

NGOs strategize to win donors’ confidence, and donors want to have confidence in their investments: Hence they fall for what Gent et al. term the “reputation trap” by donating to NGOs that have demonstrated their ability to meet measurable policy goals [10]. NGOs’ reputations can suffer if they fail to meet their long-term trajectories and focus only on goals that are quickly and visibly accomplished. The reputation trap can create a tendency among NGOs to strategically operate just like profit-seeking enterprises because of donors’ preference for funding NGOs that have developed a market image. Hence, donors tend to fund NGOs that have shown tangible success in the past, thereby generating “brands” in a sector that, paradoxically, must eschew such mundane materialistic ideas. NGO disclosure through websites can minimize the “reputation trap” and hence funding can be directed towards the NGOs that provide meaningful impact on society duly endorsed by various stakeholders. Virtual methods of monitoring NGO’s disclosures practices have the potential to circumvent the reputation trap.

This exploratory study is an effort to examine the disclosure practices followed by NGOs in Pakistan. We analyze how these practices are linked to the status of the NGO (local or international), size of the organization (estimated by number of branches), and newspaper visibility. This research conforms to the premises purported by legitimacy theory [11, 12], global governance theory [13], and globalized civil society [14]. We believe that these theories are suitable for NGOs disclosures, as they emphasize the strategies that organizations should adhere to so as to ensure that the demands of various stakeholders are met. The novelty of this study can be seen from two angles: it adds to the literature on NGOs’ disclosures, and it is generalizable to both developed and developing countries, where there is less of a tendency to regulate NGOs. This study can also help frame Standard Operating Procedures (SOPs) pertaining to online disclosures by NGOs working in Pakistan so that their contributions are justly highlighted. Consequently, NGOs and their various stakeholders, including governments, can collaborate without causing friction.

## 2. Accountability methods for websites of NGOs

A NGO’s website should provide information that makes all elements pertaining to various dimensions of accountability (i.e., professional, ethical, legal, and employment accountability, as delineated by Caulfield [15]) transparent. However, if the legal framework is ambiguous and there is no penalty mechanism, then one should expect little information on accountability. In this section, we will delineate the various approaches that have been proposed by researchers to monitor NGOs’ accountability.

### 2.1 The Boire and Prakash index

Boire and Prakash [16] proposed a non-profit accountability index to analyze the accountability practices followed by NGOs working in the United States. They [16] argued that the index they proposed covered the three important types of accountability, as defined by Ebrahim [6]. The various dimensions Boire and Prakash [16] used to construct the index are: (a) Beneficiary responsibility; (b) Codes and standards; (c) Employment responsibility; (d) Environmental responsibility; (e) Financial responsibility towards donors; (f) Public responsibility; and (g) Supplier responsibility.

Boire and Prakash [16] used IRS Form 900 to cull data on NGO’s size as measured by total revenue, percentage of government funding to a particular NGO, and service sector. They found that total revenue is negatively related to the accountability index, meaning that larger NGOs are less likely to share accountability-related information on their websites. They concluded that newspaper visibility and the accountability index are positively related, implying that NGOs that appear frequently in newspapers are more likely to share accountability-related information on their websites [16]. The percentage of government funding was found to have no association with the accountability disclosure practices followed by U.S. NGOs [16].

### 2.2 Guidance from Dumont, Rutgers e-Governance performance index and Guidestar reports

Dumont [5] measured the quality of the NGOs’ website according to three main dimensions: (a) Usability; (b) Content; and (c) Communication. The Dumont Index relied on the e-Governance Performance Index designed by the National Center for Public Performance, Rutgers University and Guidestar reports [4–5, 17–19]. Dumont [5] included following elements in each dimension of the index:

1. Usability includes the following elements: (1) Homepage length; (2) Target audience; (3) Navigation bar; (4) Site map; (5) Font color; (6) Links; (7) Site search; (8) Website update information; (9) Disability access; and (10) Languages.
2. Content includes the following elements: (1) Website copyright; (2) Location; (3) Board of Directors; (4) Contact tools; (5) Privacy statements; (6) Employee directory; (7) Mission statement; (8) Meeting minutes; (9) IRS letter of determination; (10) Annual Report; (11) By-laws; (12) Financial statements; (13) Programs and services; (14) Volunteer information; (15) Geographical Information System; (16) Calendar of events; (17) Frequently asked questions; (18) Strategic plan; (19) Performance measures; (20) Newsletter; (21) Multimedia tools; and (22) Feedback tools.
3. Communication includes the following elements: (1) Bulletin boards; (2) Responsiveness; (3) Open comments; (4) Surveys/polls; (5) Blog; and (6) Social media.

Dumont [4] used exploratory factor analysis to generate an index for NGOs’ website accountability. This index [4] was based on the dimensions previously proposed by Dumont [5]. However, in outlining these dimensions, Dumont [4] pointed out the inability of the proposed index to give weight to theoretically important elements that did not load onto the factor loadings (such as homepage length, site in more than one language, list of programs and services offered and many other elements). Factor analysis is based on factor loadings that are chosen according to maximum variability in an element, and is therefore suitable if the researcher’s intent is to analyze NGOs’ online disclosure practices. For example, if majority of NGOs do not provide a list of programs and services on their websites, it will never load onto the factors selected through factor analysis, but this does not mean that NGOs are not expected to upload this information on their websites. As the current study is an effort to provide guidelines for NGOs regarding the online accountability practices they ought to follow rather than those that the majority of the NGOs are currently following, we have used an equal weightage scheme for all the elements proposed in Section 5.

## 3. The need for a NGO disclosure index in Pakistan

Pakistan ranks 116 out of 176 countries on the Corruption Perception Index reported by Transparency International [20]. Unfortunately, the cases of dubious activities on the part of NGOs abound, and the Securities and Exchange Commission of Pakistan has revoked the licenses of 240 nonprofits under Section 42(4) of the Companies Ordinance 1984 [21]. There is no unified set of laws that governs the nonprofit sector in Pakistan; those that are applicable to NGOs include many ambiguities, and some need updating [22]. There are 17 different laws under which a social sector organization can register in Pakistan, namely: (1) The Societies Registration Act, 1860; (2) The Trusts Act, 1882; (3) The Voluntary Social Welfare Agencies Registration and Control Ordinance, 1961; (4) The Companies Ordinance, 1984; (5) The Religious Endowment Act, 1863; (6) The Charitable Endowments Act, 1890; (7) The Mussalman Wakf Validating Act, 1913; (8) The Mussalman Wakf Act, 1923; (9) The Mussalman Wakf Validating Act, 1930; (10) The Charitable and Religious Trusts Act, 1920; (11) The Income Tax Ordinance, 1979; (12) The Income Tax Ordinance, 2001; (13) The Cooperative Societies Act, 1925; (14) The Industrial Relations (Trade Unions) Ordinance, 1969; (15) The Registration Act, 1908; (16) The Charitable Funds (Registration of Collection) Act, 1953; (16) The West Pakistan Industrial and Commercial Employment (Standing Orders) Ordinance, 1968; and (17) The West Pakistan Shops and Establishments Ordinance, 1969.

The sampling framework is not available to researchers while the total number and registration statuses of such organizations are not fully known. According to the International Center for Not-for-Profit Law, NGOs are required to register with the Interior Ministry, the Ministry of Social Welfare, district governments, provincial social welfare departments, and the Department of Industries; not-for-profit companies must register with the Securities and Exchange Commission of Pakistan (SECP). As evident from the process outlined above, NGOs can register in different ways and under different authorities, and therefore there is little, if any, centralized data on these organizations. It is necessary to either synchronize all the data from the various registration authorities in a single publicly and digitally retrievable portal, or to establish a single central registration body to regulate NGOs. There is no well-structured system through which NGOs can share information; consequently, there is no mechanism for NGO disclosures through which stakeholders can digitally retrieve information on key parameters. Efforts are in progress to institutionalize the monitoring of NGOs, such as the Pakistan Center for Philanthropy (PCP), which was established in 2001 and certifies the status of NGOs; however, such assessments are voluntary—whether or not to register with the PCP is left to the discretion of the NGO. The Government of Pakistan is taking the necessary steps at a managed pace, and as a first attempt to regulate NGOs has made tax exemption status declarations by the Federal Board of Revenue conditional upon earning a certificate of good governance from the PCP [23,24].

## 4. Hypotheses to be tested

There are many theories explaining NGOs’ interest in efficient disclosure mechanisms. Shared principles and strategies have the power to form regional blocs. The Treaty of Westphalia (1648) and the Vienna Convention (1961) that made it possible for the states of diverse sizes, religions, and culture to cluster together under the European Union [25]: However, although the influence of such treaties is unquestionable, it was only after the spread of cheap communication technologies in the late 1990s that theories such as Global Governance (GG) were developed. GG is a movement that propagates collaboration between transnational actors to solve issues of mutual interest. It affects NGOs by treating them as entities that encourage equality of opportunity for all members of society and that challenges the hierarchy by ensuring that any issue is driven by a horizontal network [26]. NGOs can be seen as embedded in the Internet in a manner that promotes a nonpolitical global civil society, provided that they utilize their position to win public trust. However, this noble role of NGO can only be perceived by the public if they are able to see evidence of the NGOs’ contribution to solving social issues. NGOs should utilize cyberspace to provide this information to any individual who is interested in evaluating the success of any such organization. Just like any other organization, NGOs are also expected to collaborate both nationally and internationally to offer solutions to various social issues, thus creating a globalized civil society (GCS) [26]. The theory of GCS cannot be not fully realized unless the NGOs share information about their activities and outcomes in a shared cyberspace. Therefore, NGO websites serve as a means of creating shared goals, strategies, and best practices. The legitimacy theory states that organizations seek social license to operate by complying with the set societal norms of information sharing [11, 12]. Consequent upon legitimacy theory, the theory of globalized civil society, and the theory of global governance, we developed the hypotheses mentioned below. Each hypothesis is also backed by substantial literature, which is discussed separately before each hypothesis.

Size of an NGO is an important variable, as many researchers have argued that larger NGOs are likely to disclose more accountability information on their websites [27–30]. Larger NGOs have better designed mechanisms for accountability compliance [31]. Larger NGOs have more revenue and hence they can hire more qualified staff for record keeping and dissemination. These organizations can have a better information technology department and can therefore manage a better website than a small organization. Larger NGOs are expected to have better financial health in terms of profitability, cash flow and credit availability therefore, they have the option to hire competent staff or engage a third-party for efficient management accounting which includes management of organization’s website.

Unfortunately, due to the unavailability of data on total revenue, this study relies on the number of branches of a NGO as a proxy for size. Therefore, hypotheses (1), presented below, arise from the above theory.

$$\begin{array}{l} H_{0}\;:\;\rho_{\left(B,I_{NDI}\right)}=0\\ H_{1}\;:\;\rho_{\left(B,I_{NDI}\right)}>0 \end{array}$$ 1

The descriptive form of the above hypothesis is as follows:

H_0_: There is no correlation between the number of branches of the NGO and its disclosure score.

H_1_: There is a positive correlation between the number of branches of the NGO and its disclosure score.

Due to the quick growth of international NGOs (INGOs) over the past few decades, there is an increased expectation of disclosures and scrutiny among stakeholders; hence, INGOs are expected to have better disclosure scores [32]. The Democratic Accountability theory [33] indicates that there is an increasingly strong relationship between the amount of resources possessed by an organization and its obligations to populations deprived of those resources. This theory further implies that local communities should have the ability to demand INGOs to meet accountability demands [34]. INGOs are believed to possess more resources than NGOs, and thus, according to the Theory of Democratic Accountability, INGOs are expected to score better on disclosure indices. This gives rise to the following hypotheses:

$$\begin{array}{l} H_{0}\;:\;\mu_{NDI_{NGO}}=\mu_{NDI_{INGO}}\\ H_{1}\;:\;\mu_{NDI_{NGO}}<\mu_{NDI_{INGO}} \end{array}$$ 2

H_0_: The average disclosure score is the same for NGOs and INGOs.

H_1_: The average disclosure score for INGOS is greater than the average disclosure score for NGOs.

The media plays an important role in shaping public perception [35–38], and stakeholders invest more trust in those NGOs that share more information through various media [38]. Boire and Prakash concluded that NGOs that are more often discussed in newspapers are likely to score better on accountability [16]. Hence, we developed the following hypothesis:

$$\begin{array}{l} H_{0}\;:\;\mu_{NDI_{News.Visable}}=\mu_{NDI_{News.Not.Visable}}\\ H_{1}\;:\;\mu_{NDI_{News.Visable}}>\mu_{NDI_{News.Not.Visable}} \end{array}$$ 3

H_o_: The average disclosure score for NGOs that are mentioned in newspapers is same as the average disclosure score for NGOs that are not mentioned in newspapers.

H_1_: The average disclosure score for NGOs that are mentioned in newspapers is greater than the average disclosure score for NGOs that are not mentioned in newspapers.

## 5. The proposed index

The proposed Index, abbreviated NDI, is discussed in this section.

### 5.1 The NGO disclosure index (NDI)

The NDI relies on Dumont’s index; however, it has been redesigned specifically to suit NGOs working in lower-middle-income countries [39]: Some of the components Dumont used have been discarded, while other new components have been added [4,5]. Although Dumont [4,5] provide the major guidelines for NDI however, it will be unjust if it is not mentioned that Boire and Prakash [16] provide motivations for some of the components of NDI such as availability of annual report and audited financial statements on the website of a NGO.

The relevant literature discussed below provides sufficient support for addition of the new components:

“Clickability” has been declared an important trait that enhances the functionality of a website, and many researchers have mentioned this attribute in discussions of usability [40, 41]. Cue theory suggests that due to the pattern recognition tendency of the human brain, we like it whenever we see a clickable link, because the brain receives a message that clicking it will reveal more information [42].

The World Wide Web Consortium (W3C) is an international community that develops standards to ensure improvements in the World Wide Web. The Web Content Accessibility Guidelines (WCAG) is set of compliance standards developed by the W3C that make the targeted websites easier to use and navigate. It is used to evaluate the accessibility of any website according to four principles: (i) Perceivability; (ii) Operability; (iii) Understandability; (iv) and Robustness. The attribute of “perceivability” implies that the user interface and information must be synchronized according to a user’s perception, and users should be able to access the information through at least one of their senses (e.g., through sight, touch, or hearing). “Operability” implies that the user interface should not require any interaction that a user may not perform with ease. Hence, this trait demands accessibility features such as the provision of a keyboard emulator or a speech input mechanism. “Understandability” implies that websites should not behave in a manner that remains unpredictable. A typical example of unpredictability is if a website continues to refresh or change color; this can impede the user’s expectation of the website’s ease of use. “Robustness” implies that the user interface must not fail to support and function with changing technologies. A typical example of robustness is the ability of a website to perform well across different web browsers. WCAG 2.0 has been used by researchers to measure the quality of a website in terms of how easy it is for people with disabilities to use [43–47].

Sharing clickable links with other organizations is implicitly linked to the likelihood of collaboration between NGO and other organizations. Inter-organization collaboration increases the visibility and political standing of an organization [48]. Collaboration between local NGO and multinational enterprises can help those enterprises adapt to local needs and adjust business models [49]. Copyright information is an essential element in ranking the currency of a website, and many researchers have included it when ranking different websites [50,51]. Yazdi and Deshpande evaluated international library association websites using 15 criteria, including contact information, site maps, copyright, and other features [50]. Websites are generally developed by freelance web developers, and they reuse free developing codes that create potential perils, including identity theft, hacking, privacy infringements, and many other related issues [52]. To mitigate such perils, freelance website developers must be held accountable if the websites they develop fall prey to any of these problems, but this is only possible if the name of the website developer is known. The provision of data on past and current vacancies in any NGO can help users to comment on the genuineness of the organization. If such information is available, it reduces the likelihood that the organization is fake. In the absence of a central hub of NGOs and the existence of a string of NGO registration laws in Pakistan, it is important that NGOs provide identity information on their websites. This will help NGO registration authorities check whether a website has falsely declared itself as registered. If any NGO has been accredited by a credible national or international organization, it can help decide whether the status of the NGO is reliable or dubious. This is one method by which the Government of Pakistan was able not only to sort the good NGOs from the bad, but also help trace any so-called international organizations that recognized ghost NGOs. If a NGO declares that it has opened branches outside its headquarters, it is imperative that the addresses of any such branches are shown so that visitors to their website can use the services in the location nearest to them. It can also help funders analyze the footprints of NGOs all over the country. This information can also help the Government of Pakistan develop a central hub of information where various branches of a particular NGO are given a code that links these in a single register of information.

#### 5.1.1 NDI elements*

Elements of usability includes 11 components: Clickability, home page length, external links to local organizations, external links to international organizations, a search tab, all pertinent text on the site appearing in more than one language, site map, perceivability, operability, understandability, and robustness. The elements of content include the following 32 components: Copyrights, the name of the website developer, physical address in Pakistan, mission statement, NGO goals and strategic plan, the organization’s background information, a link for donations, past projects, list of programs, volunteer information, jobs data and jobs application portal, location shown on GIS information system (e.g., MapQuest, Google Maps, etc.), calendar of events, frequently asked questions, use of other media to educate visitors about the organization, performance measures, annual reports, audited financial statements, privacy policy, members of board of directors, minutes of board of directors, employees directory, method to apply for programs/services/ membership, community updates, newsletter availability, feedback mechanism, charity or registration number, statistical impact, reference made to the law under which the NGO is registered, NGO bylaws discussed, certification or awards, and branch addresses (in Pakistan). The NDI includes three dimensions of communication: A blog, social networking sites, and an employee and/or beneficiary survey.

*Details about calculation of the index can be obtained from the corresponding author.

## 6. The case of NGOs working in Pakistan

In this section, we will discuss the practical application of the proposed NDI.

### 6.1 The practical applications of the proposed NDI

We selected 199 NGOs active in Pakistan and created the following sampling scheme. Stratified random sampling was applied with proportional allocation, where we treated the service(s) offered by the civil society organization as the stratification variables. However, we had to estimate the strata sizes before we could apply the proportional allocation method, because there is no single and consolidated list of NGOs available in Pakistan. Therefore, we relied on a simple random sampling method to estimate the strata sizes by drawing a random sample of 954 civil society organizations and observing the relative shares of various sectors, i.e., education, health, both health and education, development, religion, and others. No holistic sampling frame was available, so various directories were utilized to select this random sample. Focusing on an estimation of the sample size, this study used a simple random sampling method of strata estimation:

1. Of the 954 randomly selected organizations, roughly 21 percent worked on the theme of education, 20 percent on health, 34 percent on both health and education, 8 percent on the development agenda, 4 percent on human rights or advocacy only, and 13 percent on other themes.
2. The total number of NGOs working in Pakistan is not known; however, according to Transparency Watch Organization, which further refers to a report by the United Nations Development Program, there are 25,000 to 35,000 [53]. If we estimate the total number of civil society organizations as 30,000 (as a median estimate), we get N = 30,000, and hence our sample size of 199 is approximately 7 percent of the total number.
3. We then estimated strata sizes by multiplying the proportions observed in step (1). The number of NGOs serving the education sector was 30,000*0.21 = 6,300; the health sector was 6,000; both the health and education sectors was 10,200; the development agenda was 2,400; human rights and advocacy was 1,200; and other types was 3,900. Finally, the sample size for each strata was determined by dividing its size by 30,000.

### 6.2 Descriptive analysis of the three dimensions of the NDI

This section covers the descriptive analysis of usability, content, and communication.

#### 6.2.1 Descriptive analysis of content

A majority of the sampled NGOs report copyright information on their websites: Almost 79 percent of the NGOs were concerned about unfair use of the information they provide publicly. Of the 199 sampled NGOs, 44 percent mentioned the name of their website developer. It is also a serious concern that 21 percent of the sampled NGOs did not provide information about their physical address in Pakistan. Furthermore, fewer than half of the sampled NGOs provided a proper mission statement on their websites. While 21 percent provided this information in a less than optimal manner, 30 percent made no effort at all in this regard. Only 53 percent of the NGOs shared proper information about their history and background. Almost 29 percent of NGOs had a donations link on their websites. It is pleasing to note that 2 percent of NGOs facilitate donations by offering multiple means of giving money, and these NGOs can serve as trend-setters for the others to follow suit. There is a lot of room for improvement regarding this criterion, as 71 percent of the sampled NGOs either did not have a donations link or had one that was not active; in some cases, the link led only to bank account information that could be used to make donations in person.

Almost 33 percent of the sampled NGOs did not share any information about the projects completed by NGOs in the past; almost 56 percent shared detailed information, while 11 percent did share information but in a vague manner (tending to discuss generic information without referring to particular projects). Almost 31 of the NGOs either did not provide any list of programs, or failed to do so in a clear manner. It was encouraging to see that as much as 69 percent of the NGOs provided sufficient information about their programs, although it was less pleasing that 66 percent of the sampled NGOs shared no information at all about volunteering. Seven percent shared some sort of description, but provided no information on how to volunteer, while 27 percent provided full information on how to and for which activity to volunteer. With regard to employment, almost 57 percent of NGOs shared no data on jobs, 8 percent had a link for jobs but the section was blank, and almost 35 percent failed to provide any proper information on current or past job openings. Almost 62 percent of the NGOs did not provide a map showing the location of their office, 7 percent showed either a static or a dynamic (i.e., featuring a zoom option) map, and almost 31 percent provided a map linked to Google Maps.

As many as 63 percent of NGOs either did not have a calendar of events on their website or included one that failed to load properly on the page and thus could not be viewed; 37 percent showed this information either implicitly or as a separate section. As many as 74 percent of the NGOs offered no information on FAQs, which may indicate either that the website users do not interact and there is no culture of asking questions, or that NGOs are not responsive to the questions being asked. Almost 74 percent of the NGOs used at least one of three modes (pictures, print media references, and electronic media references) to educate visitors to their website about their activities, while 27 percent of NGOs used all three modes. Almost 75 percent of the NGOs offered no information on the performance measures that they use to analyze their efficiency. As many as 62 percent of the NGOs did not upload annual reports on their website, while roughly 24 percent provided an archive of annual reports. Almost 76 percent of the NGOs provided no information on audited financial statements. Eighty eight percent of NGOs did not provide any information on their privacy policy.

It was quite alarming to see that 95 percent of the NGOs either had no information on their board of directors, or displayed only the directors’ names, while 96 percent either provided no information about their employees at all, or again only a list of names. It was also troubling that 67 percent of the sampled NGOs provided no information on their programs, services, or membership (PSM). The share of NGOs that displayed information on methods of applying for at least one of the three PSM activities was 33 percent. As much as 62 percent of NGOs did not provide the facility to view online or order the newsletter via email, while only 25 percent provided this information readily on their website and only 10 percent responded to email requests for newsletters. Almost 49 percent of the NGOs provided an option to provide feedback, while another 40 percent provided an email address for a person to contact in case of any concern; however, on trial submissions of feedback, about 3 percent of websites showed error messages and feedback could not be sent after three attempts to do so at random times of day. A considerable percentage, i.e. 56 percent, of the NGOs failed to display their registration number on their website. Approximately 37 percent of the NGOs did not share any statistical information about their impact, while 19 percent did so haphazardly, across different sections of their website. Approximately 40 percent of the NGOs shared information about the law under which they are registered. Only 13 percent of NGOs discussed their bylaws—a perilously low percentage. Approximately 20 percent of the NGOs shared information about certifications and awards from international NGO watchdogs. Only 27 percent of the NGOs provided information on branch offices spread across Pakistan.

#### 6.2.2 Descriptive analysis of usability and communication

The majority of the NGOs reported copyright information. Almost 79 percent of the NGOs were concerned about the unfair use of the information they provide publicly through their websites. Only three websites had fewer than three clickable links, while 25 percent of the NGOs had clickability between 0 and 9, 25 percent between 10 and 15, 25 percent between 16 and 22, and the remaining 25 percent had a clickability greater than 22. Almost 60 percent of the websites required three or more screens to be navigated in order to view the homepage of the NGO. Unfortunately, 68 percent NGOs featured no links on their websites, while only 12 percent displayed more than six links to other local organizations. Again, it was unfortunate that 53 percent of the NGOs featured no links shown on their websites, while 30 percent displayed at least six and only 10 percent displayed more than 14 links. More than half of the NGOs provided no search tab. Almost 74 percent of the NGOs did not provide any material in a language other than English, 15 percent provided material in other languages (excluding the regional languages of Pakistan), and 2 percent provided material in Urdu or a regional language where the NGO is working. Only 9 percent provided the option of viewing the website’s full contents in any other language.

As many as 90 percent of the NGOs’ websites lacked a site map. According to WCAG 2.0 guidelines, perceivability is one of the traits of an efficient website [43]. It is good that the performance of the sampled websites was sufficient, as 64 percent of the websites secured at least a B grade in this category. The second trait of WCAG 2.0 is operability; the performance of the NGOs was again sufficient in this regard, as 60 percent of the websites secured at least a B grade in this category as well. The third trait of WCAG 2.0 is understandability, but in this case the performance of the NGOs needs attention, as only 37 percent of the websites secured at least a B grade in this category. This was also the case for the fourth trait of WCAG 2.0, robustness, for which the performance of the NGOs also needs attention, as only 32.8 percent of the websites secured at least a B grade in this category.

Almost 48 percent of the NGOs had blogs on their websites. Almost 35 percent of the NGOs provided a link to their pages on at least four social networking sites. Almost 89 percent of the NGOs did not share any survey related to employees and/or beneficiaries.

#### 6.2.3 Descriptive analysis of NDI

Now, if we assume that *P*_*U*_ = *P*_*C*_ = *P*_*Com*_ = 0.5, we get the following values of weights: *W*_1_ = *W*_2_ = *W*_3_ = 0.3333. Hence, the overall NDI has scores of 42 and 0 for its maximum and minimum, respectively. One can see in Table 1 that the maximum score for the NDI is 31.5, while only 25 percent of the NGOs scored greater than or equal to 20.99, and 25 percent scored less than 12.16. The median score was 16.33.

**Table 1:** Descriptive statistics for the NDI.

N	Valid	199
	Missing	0
Percentiles	25	12.1655
	50	16.3317
	75	20.9979

## 7. Results

In Table 2, one can see that the correlation between “number of areas of operation” and the NDI is positive and significant; hence, one can say that NGOs that have more operational areas are better at sharing accountability information on their websites.

**Table 2:** Correlation between NDIs and number of areas of operation.

		Number of areas of operation	NDI
Number of areas of operation	Pearson Correlation	1	.310*
	Sig. (2-tailed)		.000
	N	199	199

* Correlation is significant at the 0.01 level (2-tailed).

In Table 3, we can see that the average score of the NDI for INGOs is 5.75 units higher than that of NGOs. The significance value for hypothesis 2 is 0.000, with a positive mean difference; hence, it is expected that the average population score of the NDI for INGOs will be greater than the average NDI for NGOs.

**Table 3:** Descriptive statistics for INGOs and NGOs.

	Status	N	Percentage	Mean	Std. Deviation
NDI	INGO	36	18.09	21.4145	6.92711
	NGO	163	81.90	15.6631	5.26092

In Table 4, we can see that almost 65 percent of the NGOs are featured in newspapers, while 35 percent are not. The sample average of NGOs that are not featured in newspapers is 3.44 units lower than that of those that are featured. The significance value for hypothesis 3 is 0.000, with a negative mean difference; thus, the average NDI for NGOs that are not featured in newspapers is lower than that of NGOs that are.

**Table 4:** Descriptive statistics for being featured in newspapers.

	Newspaper visibility	N	Percentage	Mean	Std. Deviation
NDI	No	70	35.2	14.4724	6.10736
	Present	129	64.8	17.9142	5.60926

The role of the media was found to be positive, as NGOs that are featured in newspapers have yielded better disclosure scores than those that are not. There were fewer information asymmetries for NGOs with branches, as they scored better on the disclosure index. The best performances in the content category were for “Use of Other Media,” “List of Programs,” and “Feedback Mechanism,” with compliance percentages of 95, 91.4, and 91, respectively. The worst performances in the content category were for “NGO Bylaws,” “Privacy Policy,” and “Board of Directors Minutes,” with compliance percentages of as low as 12.6, 12.1, and 0, respectively. The best performances in the communication category were for “Clickability,” “Perceivability,” and “Operability,” with compliance percentages of 97, 64, and 60, respectively. The worst performances in the communication category were for “Site in More than One Language,” “External Links to Local Organizations,” and “Site Map,” with compliance percentages of as low as 26.1, 18.6, and 10.1, respectively.

The NDI was dichotomized using the sample median, i.e., all values lower than 16.33 were recoded as zero and all other values were recoded as one. A logistic regression was then applied, the results of which are presented in Table 5 below. This model performed very well, as the it predicted the NDI correctly almost 70 percent of the time. Cases where the NDI = 0 were predicted with 80 percent accuracy, while NDI = 1 cases also met the minimal criterion of 60 percent accuracy. In Table 5 below, one can see that the odds ratio for NGOs with branches is 1.760, meaning that there are 1,760 such NGOs whose NDI = 1, compared to 1,000 single-office NGOs whose NDI = 1. In other words, the odds of having better disclosure scores are 1.76 times higher for NGOs with branches, compared to single-office NGOs. Similarly, the odds of NGOs having a better disclosure score are almost half that for INGOs. Finally, the odds of obtaining a better disclosure score for NGOs that are featured in newspapers are 1.57 times higher than that for those that are not featured in newspapers.

Wald test has been used to check the significance of each variable while 5% level of significance has been used to declare a variable as significantly related to NDI. Note that Wald test cannot be used to check the overall goodness of the model or the collective ability of the independent variables to explain the dependent variable. For such purpose, one can use the Hosmer-Lemeshow test that calculates the percentage of cases correctly predicted by the model. This model performed very well, as the model predicted the NDI correctly almost 70 percent of the time. Cases where the NDI = 0 were predicted with 80 percent accuracy, while NDI = 1 cases also met the minimal criterion of 60 percent accuracy. Given that this model is newly constructed and an NGO disclosure is a sensitive variable, these scores demonstrate an excellent degree of accuracy.

**Table 5:** Model summary logistic model of the NDI.

	B	df	Sig.	Exp(B)
Branches	.565	1*	.000	1.760
NGOs	-.794	1	.000	.452
Newspaper coverage	.454	1	.000	1.574
Constant	.565	1	.228	1.759

*Note that the significance of each variable is tested using the Wald test that follows Chi-square distribution with degrees of freedom fixed at one [54].

## 8. Future research

The proposed index is based on set of criterions that should be followed by the NGOs in a country where practices of efficient website management are not necessarily followed by NGOs. This is the reason the proposed index avoids use of exploratory factor analysis (EFA) due to the inability of this method to weigh elements that are theoretically important but not generating sufficient in-sample variations. However, one must keep in mind that such techniques should be applied later when NGOs are monitored as a rule by some watchdog body and hence, the NGOs focus on efficient flow of information on its website. In other words, for the case of Pakistan one can expect that application of EFA, after five to ten years, can produce promising results for the stakeholders.

The calculation of the proposed index needs assessment of the NGOs’ websites which if done manually can be cumbersome. Hence, future researches can focus on making use of web crawling technology or a computer program in software such as *Rcrawler* to automatically calculate this index.

## 9. Conclusion

This study focused on designing an online disclosure index for NGOs working in lower-middle-income group countries. The proposed index is by no means final; it is a preliminary tool that can be improved by researchers. The index has been calculated for use in Pakistan: Although international NGOs are subject to increased monitoring by the Government of Pakistan, it has been demonstrated that such indices encourage better accountability compliance.

The NDI is a holistic compliance kit for NGOs. If these organizations choose to follow the proposed framework, it can help them earn the trust of the public and a vote of confidence from the government. There is a serious need to provide evidence of the synergy impact created by various NGOs, as this information is lacking on the websites of NGOs analyzed. Compliance according to the proposed index can increase the likelihood of international funding for NGOs, as many local initiatives can be funded by reputable international missions provided that they meet certain criteria, many of which are present in the NDI. The altruistic nature of NGOs can be better portrayed in the international community if evidence of the benefit to society is readily available, in multiple languages, on the website of each NGO. Given the fact that there has been a concern internationally that NGOs should comply with laws, the NDI inclusiveness of bylaws can serve as a guarantee that government and watchdog organizations will view these NGOs positively. The NDI should be evaluated on a continuous basis, and PCP certification may be linked with a good score on this index along with other criteria. Stakeholders may be contacted about the usefulness of the index, and donors may be asked whether they can trust it if it is evaluated by neutral agents, such as the authors of the current study.

## Appendix I

### A mathematical framework for the NDI

In this section, we have discussed the mathematical formulation of the proposed NGOs disclosure index for NGOs. This analysis will help us optimize the index subject for the typical statistical practice of designing estimators with minimum variation.

Let *Y* = *W*_1_*X*_*U*_ + *W*_2_*X*_*C*_ + *W*_3_*X*_*Com*_, where *W*_1_ + *W*_2_ + *W*_3_ = 1 and *W*_*i*_, *I* = 1,2,3 are weights assigned to the NDI on an optimal variance basis. Let us also suppose that *I*_*NEAI*_ = *W*_1_*I*_*U*_ + *W*_2_*I*_*C*_ + *W*_3_*I*_*Com*_ Note that *X*_*U*_,*X*_*C*_ and *X*_*Com*_ are each Bernoulli random variables with $p_{i}=p\left(I_{j}>{\tilde{I}}_{j}\right)$, where *j* = *U*, *C*, *Com* and ${\tilde{I}}_{j}$ is the minimum acceptable standard for the *j*^*th*^ dimension of the NDI. In other words, $X_{j}=\left[\begin{array}{ccc} 0 & if & I_{j}\leq {\tilde{I}}_{j}\\ 1 & if & I_{j}>{\tilde{I}}_{j} \end{array}\right],\;j=U,C,Com$.

Let us define *I*_*ji*_ as the score on the *j*^*th*^ dimension of accountability for the *i*^*th*^ NGO, where *i* = 1,2,….*n* and *j* = *U*, *C*, *Com*. Let us also define the overall index of accountability for the *j*^*th*^ dimension as follows; $I_{j}=\sum_{i=1}^{n}\sum_{l=1}^{k_{j}}I_{jil},\;j=U,C,Com$, *i* represents *i*^*th*^ NGO and *l* is used to express variation in criteria for the *j*^*th*^ dimension of accountability, while *k*_*j*_ represents the number of criterions in the *j*^*th*^ dimension. Furthermore, observe that 0 ≤ *I*_*j*_ ≤ 3*k*_*j*_ because the maximum score for each criterion is 3. (Clickability = *I*_*Ui*1_, Home Page Length = *I*_*Ui*2_,….. Robust = *I*_*Ui*(11)_). Now we can safely assume that each of the *I*_*jil*_ follows a Bernoulli distribution, with the probability of success equal to *p*_*j*_ i.e. *I*_*Ui*1_ ∼ *B*(*p*_1_), *I*_*Ui*2_ ∼ *B*(*p*_2_), *I*_*Ui*3_ ∼ *B*(*p*_3_),……*I*_*Ui*(11)_ ∼ *B*(*p*_(11)_). According to the Poisson Binomial Distribution, Le Cam’s inequality indicates that the distribution of *I*_*j*_ is Poisson with *λ*_*j*_, where $\lambda_{j}=\sum_{l=1}^{k_{j}}p_{l}$ [55–57]. Now the probability that *X*_*j*_ = 0 is $p\left(I_{j}\right)=I_{j}\leq {\tilde{I}}_{j}=\sum_{Y=0}^{{\tilde{I}}_{j}}\frac{e^{-\lambda_{j}}{\left(\lambda_{j}\right)}^{Y}}{Y!}$. Furthermore, $V\left(Y\right)=W_{1}^{2}V\left(X_{U}\right)+W_{2}^{2}V\left(X_{C}\right)+W_{3}^{2}V\left(X_{Com}\right)$, where use has been made of the fact that *X*_*j*_s are mutually independent. Now, relying on the fact that the variance of a *j*^*th*^ Bernoulli random variable is *p*_*j*_*q*_*j*_, we can write that:

$$\begin{array}{l} V\left(Y\right)=W_{1}^{2}A+W_{2}^{2}B+W_{3}^{2}C,\;A=\left(\sum_{Y=0}^{{\tilde{I}}_{1}}\frac{e^{-\lambda_{1}}{\left(\lambda_{1}\right)}^{Y}}{Y!}\right)\left(\sum_{Y={\tilde{I}}_{1}}^{3k_{1}}\frac{e^{-\lambda_{1}}{\left(\lambda_{1}\right)}^{Y}}{Y!}\right),\;B=\left(\sum_{Y=0}^{{\tilde{I}}_{2}}\frac{e^{-\lambda_{2}}{\left(\lambda_{2}\right)}^{Y}}{Y!}\right)\left(\sum_{Y={\tilde{I}}_{2}}^{3k_{2}}\frac{e^{-\lambda_{2}}{\left(\lambda_{2}\right)}^{Y}}{Y!}\right)\\ C=\left(\sum_{Y=0}^{{\tilde{I}}_{3}}\frac{e^{-\lambda_{3}}{\left(\lambda_{3}\right)}^{Y}}{Y!}\right)\left(\sum_{Y={\tilde{I}}_{3}}^{3k_{3}}\frac{e^{-\lambda_{3}}{\left(\lambda_{3}\right)}^{Y}}{Y!}\right).\;\mathrm{As}\;W_{1}+W_{2}+W_{3}=1,\;\mathrm{therefore}\\ V\left(Y\right)=W_{1}^{2}A+W_{2}^{2}B+{\left(1-W_{1}-W_{2}\right)}^{2}C. \end{array}$$

Optimizing the variance expression w.r.t *W*_*j*_ yields optimum weights.

$$\frac{\partial }{\partial W_{1}}\left[V\left(Y\right)\right]=2W_{1}A+2\left(1-W_{1}-W_{2}\right)(-1)C=0$$ (4)

$$\frac{\partial }{\partial W_{2}}\left[V\left(Y\right)\right]=2W_{2}B+2\left(1-W_{1}-W_{2}\right)(-1)C=0$$ (5)

From (4) we can write:

$$W_{1}=\frac{C\left(1-W_{2}\right)}{A+C}$$ (6)

Using (5) and (6) we get:

$$W_{2}=\frac{CA}{BA+BC+BC}=\frac{1}{1+\frac{B\left(A+C\right)}{CA}}=\frac{1}{1+\frac{V(X_{C})\left[V\left(X_{U}\right)+V\left(X_{Com}\right)\right]}{V\left(X_{U}\right)V\left(X_{Com}\right)}}$$ (7)

Therefore, (6) and (7) imply that:

$$W_{1}=\frac{BC}{BA+BC+BC}=\frac{1}{1+\frac{A\left(B+C\right)}{BC}}=\frac{1}{1+\frac{V(X_{U})\left[V\left(X_{C}\right)+V\left(X_{Com}\right)\right]}{V\left(X_{C}\right)V\left(X_{Com}\right)}}$$ (8)

Therefore, the optimum variance of *I*_*NDI*_ becomes:

$$\begin{array}{l} V\left(Y\right)={\left[\frac{1}{1+\frac{V(X_{U})\left[V\left(X_{C}\right)+V\left(X_{Com}\right)\right]}{V\left(X_{C}\right)V\left(X_{Com}\right)}}\right]}^{2}V(X_{U})+{\left[\frac{1}{1+\frac{V(X_{C})\left[V\left(X_{U}\right)+V\left(X_{Com}\right)\right]}{V\left(X_{U}\right)V\left(X_{Com}\right)}}\right]}_{}{}^{2}V(X_{C})+\\ {\left[1-\left[\frac{1}{1+\frac{V(X_{U})\left[V\left(X_{C}\right)+V\left(X_{Com}\right)\right]}{V\left(X_{C}\right)V\left(X_{Com}\right)}}\right]-\left[\frac{1}{1+\frac{V(X_{C})\left[V\left(X_{U}\right)+V\left(X_{Com}\right)\right]}{V\left(X_{U}\right)V\left(X_{Com}\right)}}\right]\right]}_{}{}^{2}V\left(X_{Com}\right) \end{array}$$ (9)

Note that for a fixed $V\left(X_{C}\right)\;\mathrm{and}\;V\left(X_{Com}\right)\;\mathrm{as}\;V\left(X_{U}\right)↑\left[\frac{V(X_{U})\left[V\left(X_{C}\right)+V\left(X_{Com}\right)\right]}{V\left(X_{C}\right)V\left(X_{Com}\right)}\right],↓\;\mathrm{consequently}\;W_{1}↓$.

**Fig 1:**
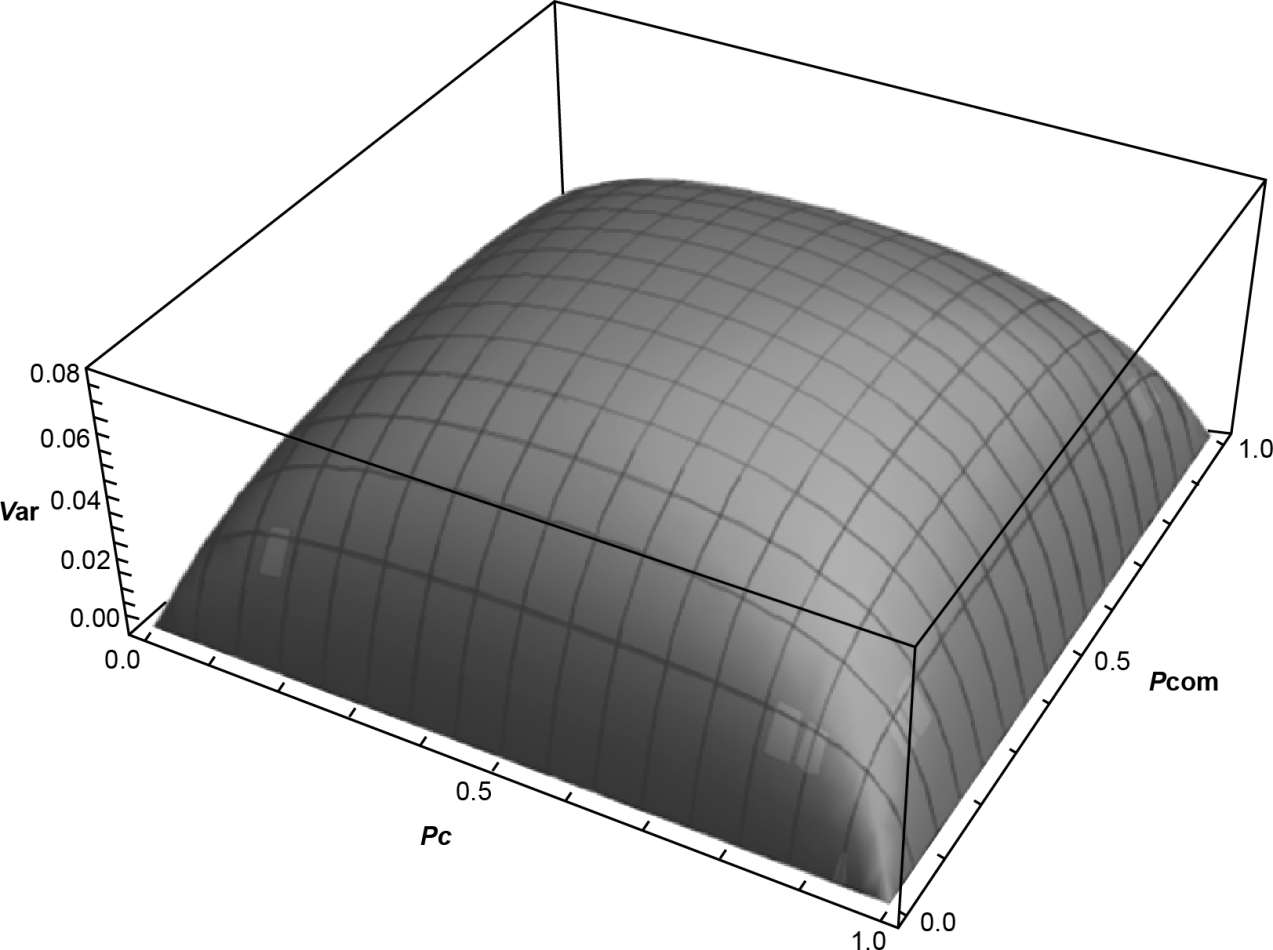
Three-dimensional plot of variance expression for the NDI.

From a simulation perspective, we can let (*p*_*U*_,*q*_*U*_),(*p*_*C*_,*q*_*C*_) and (*p*_*Com*_,*q*_*Com*_) vary over (0,1) and observe the patterns in the values of *V*(*I*_*NEAI*_). Fig 1 shows the response of *V*(*I*_*NEAI*_) to changes in either of *p*_*U*_,*p*_*C*_ or *p*_*Com*_. This pattern shows that *V*(*I*_*NEAI*_) is an increasing function of *p*_*j*_ until 0.5, after which *V*(*I*_*NEAI*_) begins to decrease and there is an accompanying increase in *p*_*j*_. The meaning of $P\left(I_{j}>{\tilde{I}}_{j}\right)>0.5$ is that more than 50 percent of the NGOs meet the *j*^*th*^ threshold for the NDI. Hence, if more than 50 percent of the NGOs meet the *j*^*th*^ threshold, then the variability in *V*(*I*_*NEAI*_) starts decreasing.

## Supporting information

S1 File (SAV)
